# Recovery of H14 influenza A virus isolates from sea ducks in the Western Hemisphere

**DOI:** 10.1371/currents.RRN1290

**Published:** 2012-02-02

**Authors:** Jacqueline Nolting, Anthony C. Fries, Richard D Slemons, Chad Courtney, Nichole Hines, Janice Pedersen

**Affiliations:** ^*^Ohio State University Type A Animal Influenza Laboratory Research Associate; ^†^The Ohio State University Department of Evolution, Ecology, and Organismal Biology Graduate Research Associate; ^‡^Veterinarian, Department of Veterinary Preventive Medicine, The Ohio State University; ^§^Ohio State University, Department of Veterinary Preventive Medicine, Research Associate; ^¶^United States Department of Agriculture, National Veterinary Services Laboratories and ^#^Microbiologist Avian Section Diagnostic Virology Laboratory, National Veterinary Services Laboratories, Veterinary Sevices, United States Department of Agriculture

## Abstract

In 2010, H14 influenza A viruses were recovered from clinically normal sea ducks in the United States. These are the first H14 isolates recovered in the Western Hemisphere and represent the only documented H14 influenza A viruses isolated since the original isolates were recovered from near the Caspian Sea during 1982.

## Introduction

It is well established that wild waterfowl, shorebirds, and gulls are natural reservoirs for antigentically diverse populations of influenza A viruses which include all known influenza A virus hemagglutinin (HA) and neuraminidase (NA) subtypes [1] [2] [3] [4]. Of the thousands of avian-origin influenza A virus (AIV) isolates previously recovered around the world, only three or four isolates have possessed the H14 HA subtype and these isolates were recovered from wild birds in the Eastern Hemisphere. More specifically, these Eastern Hemisphere H14 isolates were recovered from a herring gull (*Larus argentatus*) and wild mallards (*Anas platyrhynchos*) sampled just north of the Caspian Sea [5]. The genomic sequences for the hemagglutnin gene of these original H14 isolates appear in GenBank.  

During the fall waterfowl migration of 2010, routine virus surveillance efforts in the Mississippi Migratory Bird Flyway resulted in the recovery of three additional H14 influenza A virus isolates, two from long-tailed ducks (*Clangula hyemalis*) and one from a white-winged scoter (*Melanitta fusca*). Nucleotide sequencing was done on the HA gene of these new isolates to confirm the antigenic sub-typing results and to compare their nucleotide and amino acid sequences to the Caspian Sea H14 AIV isolates published in GenBank.   

## **Study**

      From September 2010 through January 2011, during the fall waterfowl hunting season, a total of 2,870 cloacal swabs or environmental fecal samples were collected from hunter-harvested dabbling, diving, and sea ducks sampled at 35 study sites located in nine states in the Mississippi Migratory Bird Flyway in the United States. (U.S Fish & Wildlife Service permit #MB219513-1). These samples were collected as part of our ongoing AIV surveillance program sponsored by the Minnesota Center for Excellence for Influenza Research and Surveillance (MCEIRS). Virus isolation attempts on each sample were done using 10-day-old specific-pathogen free embryonating chicken eggs [6]. All chroio allantoic fluids with hemagglutinating activity were tested for the presence of influenza A virus using the Avian Influenza Virus Type A Antigen Test Kit (Synbiotics Corporation, San Diego, CA) . A total of 145 AIV isolates were recovered (5.1% of samples). These AIV isolates were submitted to the USDA, APHIS, National Veterinary Services Laboratories (NVSL) in Ames, Iowa for HA and NA sub-typing using standard hemagglutination-inhibition (HI) and neurimindase-inhibition (NI) tests, respectively. Among these 145 AIV isolates, the NVSL HI test identified three H14 isolates and their associated NA subtypes (N6 and N4,8) as well as numerous other HA-NA combinations common to waterfowl in North America. The N4,8 antigenic subtyping possibly represents a mixed infection or cross reaction in the NI test.

      The H14 positive isolates were all recovered from samples collected at the same location in Wisconsin. The isolates are identified as A/long-tailed duck/WI/10OS3912/2010 (H14N6) and A/white-winged scoter/WI/10OS3922/2010 (H14N4,8), which were recovered from samples collected on November 16, 2010, and A/long-tailed duck/WI/10OS4225/2010 (H14N6) from a sample collected on December 3, 2010.

      In order to confirm the antigenic HA subtype of our three H14 isolates, DNA sequencing was performed to compare their HA nucleotide sequences to all AIV HA genomic sequences found in GenBank. Briefly, reverse transcription was done using AMV reverse transcriptase per the manufacturers instruction (Thermo Scientific Cat #F-5705). PCR was performed using previously published H14 specific primers [7] with the Paq5000 Hot start master mix (Agilent cat# 600870). Target size for the H14 HA was approximately 550 base pairs, which was confirmed by gel electrophoresis. 

      For HA gene sequencing, the BigDye Terminator v.3.1 cycle sequencing kit (Applied Biosystems #4337455) was used for Sanger sequencing of fragments using the same forward and reverse primers as those used for cDNA amplification.  Cycle-sequenced fragment reactions were cleaned using Sephadex G-50 (Sigma Aldrich #G5080) and Centri-Sep collection columns (Princeton Separations).  Samples were run on an ABI Prism 3100 Genetic Analyzer and subsequently analyzed for quality of forward and reverse sequences using CodonCode Aligner (v. 3.7.1 Codon Code Corporation). Sequences were aligned using the Clustal W [8] application in BioEdit v. 7 [9].   MEGA5 [10] was used for sequence analysis and maximum likelihood (ML) tree construction based on 1000 bootstrap replications and a GTR+I+G model of nucleotide evolution as selected by jModelTest [11]. Sequences obtained were submitted into GenBank, accession numbers JN696314-JN696316.

        We recovered 551 base pairs from each of our three H14 HA genomic segments.  One polymorphic site was found between the three isolates at position 314 of the fragment which was a synonymous nucleotide mutation. The Basic Local Alignment Search Tool (BLAST) application on National Center for Biotechnology Information (NCBI) was used to determine the closest related AIV sequences currently present in GenBank. Four AIV strains A/mallard/Gurjev/263/1982(H14N5), A/mallard/Astrakhan/263/1982(H14N?), A/Mallard/Gurjev/244/1982(H14N6), and A/herring gull/Astrakhan/267/1982(H14N5)) were identified as the closest matches to our 551 bp sequence query at 89% similarity.  Therefore, the closest matches to the three Wisconsin H14 isolates were the H14 AIVs isolates from near the Caspian Sea in 1982. There was a 93.9% (172 of 183) amino acid similarity between the 2010 and 1982 H14 translated sequences.  The HA nucleotide sequences were <72% similiar for other AIVs in GenBank with the next closest sequences being strains of H4 AIVs (Figure 1). 

**Figure:**
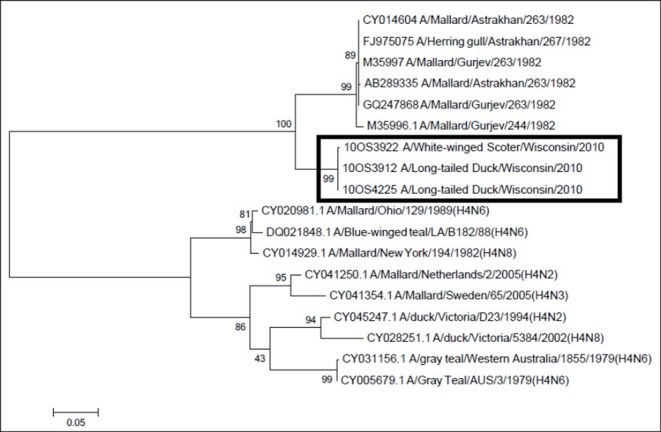
**Figure 1.  ML phylogenetic tree of HA genes of H14 viruses from the United States. **This tree includes the published nucleotide sequences of six Caspian Sea area H14 HA sequences from 1982, nine published H4 HA gene sequences which are the next most closely related nucleotide sequences to the U.S. H14 HA genes.

## **Conclusions**

      The recovery of three H14 AIV isolates from sea ducks in the Mississippi Migratory Bird Flyway was a first for North America and identified temporal and spatial gaps in our knowledge about the natural history of the H14 AIVs.  The genomic relationships between the HA genomic segments of the North American and the Caspian Sea area H14 AIV isolates recovered in Europe 28 years earlier raises the possibility of an unrecognized niche in the ecology of influenza A viruses. The undetected maintenance of H14 AIVs during the previous 28 years could be due to several biological mechanisms including, but not limited to, the following: low incidence of infections in an unrecognized host; the natural host not being included in surveillance efforts; the virus remaining in a static state in the environment; new molecular screening diagnostic protocols not including H14 AIVs, etc. 

      Thousands of wild ducks, mainly dabbling and diving ducks, are tested each year during routine surveillance in both North America and Eurasia with no H14 HA recoveries being reported since 1982. The inherent bias of sampling protocols, sampling schedules, and diagnostic methods may have contributed to the scarcity of H14 AIV isolates during this 28 year hiatus. The fact that these North American H14 isolates were recovered from sea ducks well after the departure of most dabbling and diving ducks due to frozen lakes and marshes could indicate another ecological niche or component in the natural history of AIVs in waterfowl. In 1982, one of the original H14 isolates was recovered from a herring gull. Interestingly, white-winged scoters often nest in association with gull breeding colonies ([12]). This might be a clue for examining links between species to determine the transmission cycle of the apparently rare H14 AIVs. Furthermore, it will need to be determined if these new North American H14 isolates or the Caspian Sea H14 isolates were recovered from spill-over hosts or represent the true maintenance hosts.

      Currently, sequencing of the remaining seven genomic segments is underway to gain insight into the evolutionary origin of each of the genomic segments. Determining the evolutionary history of all eight segments will hopefully provide additional clues as to the location and host species where H14 AIV isolates are being maintained.  The recovery of these H14 AIV isolates is being used to adjust our surveillance efforts to shift our focus on sea ducks to increase the likelihood of recovering additional H14 AIV isolates and gaining a further understanding of the ecology of these viruses.  

## **Funding information**

      This work has been funded in part with federal funds from the Centers of Excellence for Influenza Research and Surveillance (CEIRS), National Institute of Allergy and Infectious Diseases, National Institutes of Health, Department of Health and Human Services, under Contract No. HHSN266200700007C. Its contents are solely the responsibility of the authors and do not necessarily represent the official views of the NIH.  

## **Competing interests**

The authors have declared that no competing interests exist.
